# Biosorption of malachite green from aqueous solutions by *Pleurotus ostreatus* using Taguchi method

**DOI:** 10.1186/2052-336X-12-63

**Published:** 2014-03-12

**Authors:** Zhengsuo Chen, Hongbo Deng, Can Chen, Ying Yang, Heng Xu

**Affiliations:** 1 Key Laboratory of Bio-resources and Eco-environment (Ministry of Education), College of Life Science, Sichuan University, Chengdu, Sichuan 610064, China

**Keywords:** Malachite green, *Pleurotus ostreatus*, Biosorption, Taguchi method

## Abstract

Dyes released into the environment have been posing a serious threat to natural ecosystems and aquatic life due to presence of heat, light, chemical and other exposures stable. In this study, the *Pleurotus ostreatus* (a macro-fungus) was used as a new biosorbent to study the biosorption of hazardous malachite green (MG) from aqueous solutions. The effective disposal of *P. ostreatus* is a meaningful work for environmental protection and maximum utilization of agricultural residues.

The operational parameters such as biosorbent dose, pH, and ionic strength were investigated in a series of batch studies at 25°C. Freundlich isotherm model was described well for the biosorption equilibrium data. The biosorption process followed the pseudo-second-order kinetic model. Taguchi method was used to simplify the experimental number for determining the significance of factors and the optimum levels of experimental factors for MG biosorption. Biosorbent dose and initial MG concentration had significant influences on the percent removal and biosorption capacity. The highest percent removal reached 89.58% and the largest biosorption capacity reached 32.33 mg/g. The Fourier transform infrared spectroscopy (FTIR) showed that the functional groups such as, carboxyl, hydroxyl, amino and phosphonate groups on the biosorbent surface could be the potential adsorption sites for MG biosorption. *P. ostreatus* can be considered as an alternative biosorbent for the removal of dyes from aqueous solutions.

## Background

Dyes are widely used in textile, leather, paper, rubber, plastics, cosmetics, pharmaceutical, food plants, printing and dyeing industry in China, which consumed large quantity of wastewater from different steps in the dyeing and finishing processes. It is reported that the discharge volumes of these wastewater reaches 14.13 × 10^8^ tons per year [1]. These dyes from such industrial effluents are usually released into the environment which can be carcinogenic, mutagenic, allergenic, toxic to the aquatic life, the food web and can be resistant to natural biological degradation due to presence of these heat, light, chemical and other exposures stable [2], thus upsetting aquatic life [3]. Hence, the removal of such colored agents from aqueous effluents becomes significantly environmental, technical, and commercial importance [4].

MG is a cationic dye, an N-methylated diaminotriphenylmethane dye and has been widely used for the dyeing of leather, wool and silk, distilleries, jute, paper, cotton, leather, etc. [5], which is generally used as a strong anti-fungal, anti-bacterial and anti-parasitical agent in aquaculture [6]. The powerful antimicrobial activity of MG has been attributed to inhibition of intracellular enzymes, intercalation into DNA, and/or interaction with cellular membranes. For its high toxic, high residual, teratogenic, carcinogenic, mutagenic and other side effects to fresh water fish [7,8] and the detection of MG though foodstuff destined to human consumption, alarmed the health hazards against human being. Though the use of this dye has been banned in several countries, it is still being used in many areas due to its low cost, ready availability and efficacy [9,10]. In 2002, MG has been prohibited to be used as raw material for all food animals by Ministry of Agriculture of China, but the results of domestic market survey showed that: malachite green was still commonly used in aquiculture and aquatic products trafficking.

There are numbers of methods, like decolourisation, coagulation method, chemical oxidation, electrolytic process to eliminate dyes from wastewater. However, studies confirmed that the products formed after degradation of MG were also not safe and had carcinogenic potential [9,11]. Adsorption is widely studied and applied for its low cost, high efficiency and easy operation in recent years [12]. The removal of dyes from wastewater is considered to be an important application of adsorption process using suitable adsorbent [13]. Low cost materials such as *Prosopis cineraria*[14], Hen feathers [15], Jute stick [16], Tea leaves [17], *Tricholoma lobayense*[1], Bagasse [5] and Peanut husk [18] have been reported to be used as biosorbent for the efficient removal of dyes from aqueous solution.

In this paper, a novel biomaterial *P. ostreatus* was used to remove MG from aqueous solutions. The macro-fungus biomasses (dead, living or their derivatives) had been studied of their adsorptive properties. Previous studies have reported that they have the capacity to bind metals [19,20], dyes [21,22] or other organic pollutant [23]. The macro-fungus *P. ostreatus*, commonly known as a mushroom, is an edible basidomycete. No matter in temperate or tropical climates, *P. ostreatus* can be found easily*.* It is now the second most important cultivated mushrooms in the world [24], and is one of the most common edible mushrooms in China. Its biomass is cheap and easily available. Therefore, the purpose of this study was to evaluate the potential of *P. ostreatus* as a biosorbent to remove MG from aqueous solutions. The operational parameters such as biosorbent dose, pH and ionic strength were studied. Biosorption isotherm models and kinetics models were studied. Taguchi method was used to determine the significant factors and the optimum levels of experimental factors for MG biosorption. FTIR was used to research the characteristics of biosorbent surface.

## Methods

### Adsorbate

MG was used as a representative cationic dye in this study. It was of analytical grade chemicals (C.I. = 42,000, chemical formula = C_23_H_25_ClN_2_, FW = 364.92, λ_max_ = 617 nm) and purchased from Kelong Chemical Reagent Factory, Chengdu, China. The stock solution (1000 mg/l) of MG was prepared by dissolving accurately weighed amount of the dye in distilled water. Experimental solutions of the desired concentrations were obtained by successive diluting the stock solution by distilled water.

### Biosorbent

The macro-fungus *P. ostreatus* was purchased from a mushroom production site near Chengdu, Sichuan. The samples were repeatedly cleaned with deionized water to remove adhering dirt and soluble impurities, and boiled with distilled water three times, each for 3–5 minutes filter with the distilled water until the filtered water was cleared. They were dried at 50°C until keep constant weight, then crushed and sieved through 200 mesh (< 75 μm) sieve to get the smaller particles. The sieved biosorbent were kept in a plastic sealable bag and stored in a desiccator without any further chemical or physical treatment before adsorption experiments.

### Characterization of biosorbent

Fourier transform infrared spectrometer (FTIR) (NEXUS-650, America) was used to analyse the functional groups on the biosorbent surface, which may be responsible for MG biosorption.

The pH at the point of zero charge of the biosorbent (pH_PZC_), namely the pH value required to give zero net surface charge, was determined by mass titration. Three solutions with different initial pH values (3, 6 and 11, respectively) were prepared and with the addition of 0.05 M aqueous solution of HNO_3_ or NaOH, while NaNO_3_ were used as the background electrolyte. For each initial pH, six Erlenmeyer flasks were filled with 30 mL of the solution and different amounts of biosorbent were added (0.05%, 0.1%, 0.5%, 1%, 5% and 10% by weight). Then the mixture was shaken for 24 h at a thermostatic swing shaker. The pH was measured after the biosorbent was separated. A plot of the equilibrium pH versus mass fraction yielded a curve showing a plateau and the pH_PZC_ was identified as the point at which the change of pH is zero. The pH_PZC_ was then taken as the average of the three asymptotic pH values [22].

### Experimental methods and measurements

The concentration of MG in the solution before/after equilibrium biosorption was determined by using a double beam UV/vis spectrophotometer to find out the absorbance at λ_max_ of 617 nm. The professional data processing and mapping software Origin 8.5 was used for data analysis and graphics production. Calibration curve for MG was done before all the experiments, by the regression equation, the concentration of MG of every process could calculate precisely. The dye concentrations were measured at time t = 0 and at equilibrium, the biosorption capacity at equilibrium, *q*_*e*_ (mg/g), was calculated by using the following equation [25]:

(1) $$q_{e}=\left(C_{0}-C_{e}\right)\cdot \frac{v}{w}$$

(2) $$P=\frac{C_{0}-C_{e}}{C_{0}}\cdot 100\%$$

Where *C*_*0*_ is the concentrations of MG at initial time and *C*_*e*_ is the concentrations of MG at equilibrium, respectively (mg/L). *V* is the volume of the solution (L) and *W* is the biosorbent dose (g), *q*_*e*_ is the biosorption capacity per unit mass of biosorbent (mg/g) and *P* for the removal efficiency (%).

### Batch biosorption studies

A series of experiments were carried out in 150 ml stoppered glass Erlenmeyers flasks with 50 ml MG solution to investigate the operational parameters such as the effect of biosorbent dose, initial MG concentration, contact time, pH and ionic strength. Erlenmeyers flasks were placed at a shaker (SUKUN) with a shaking of 180 rpm and at 25°C for 6 h. After biosorption, all the samples were centrifuged with a Centrifuge (Thermo) at 10,000 g for 5 min to separate the solid phase from the liquid phase, and then analysis of the residual concentration of MG by spectrophotometer. To investigate the effect of biosorbent dose on MG removal, different weight of fungal biosorbent (varying from 0.05 g to 0.35 g) was added in each MG solution (40 mg/L). The effects of the medium pH on biosorption capacities of the biosorbent was studied from 2 to 10 (i.e. 2, 4, 6, 8 and 10), which was adjusted by adding a few drops of diluted aqueous solutions of HCl or NaOH (0.10 M). Ionic strengths of MG solutions was studied at four different NaCl (i.e. 0.05, 0.1, 0.15 and 0.2 M) and CaCl_2_ (i.e. 0.05, 0.1, 0.15 and 0.2 M). All experiments were conducted in duplicates and two controls were performed. Dye control (dye solution without biosorbent) were also shaken using the same procedure. Biosorbent control (biosorbent in double-distilled water without MG) with the same procedure.

### Biosorption isotherms and kinetics studies

Biosorption isotherm models are used to indicate the interactions between adsorbate and biosorbent. The biosorption isotherm models were analyzed using the Langmuir [26] and Freundlich [27] isotherm models to determine the mechanism of biosorption in this study. For the biosorption isotherms studies, biosorption experiments were carried out at different initial MG concentration (i.e. 20, 40, 60, 80 and 120 mg/L).

Three common kinetics models, pseudo-first-order [28], pseudo-second-order [16] and intraparticle diffusion [29] were used to study the mechanism of adsorption. For the kinetic studies, the experimental samples were taken at predetermined time intervals for analyzing the residual dye concentration. Table 1 represents the equation of all the above-mentioned models.

**Table 1:** Models equations of biosorption isotherms and kinetics studies

**Model**	**Equation**	**Symbols representation**
Langmuir isotherms model	$q_{e}=\frac{k_{a}C_{e}q_{m}}{1+k_{a}C_{e}}$	where *q*_*e*_ and *q*_*m*_ are the equilibrium and maximum adsorption capacity of MG, respectively (mg/g), *C*_*e*_ is the concentration of MG at equilibrium (mg/L), *k*_*a*_ is the Langmuir constant (L/mg).
Freundlich isotherm model	$\mathrm{In}q_{e}=\mathrm{In}K_{F}+\frac{1}{n}\mathrm{In}C_{e}$	where *K*_*F*_ (L/g) and n are Freundlich isotherm constants which related to biosorption capacity and biosorption intensity, respectively.
Temkin isotherm model	*q*_*e*_ = *B*In(AC_*e*_)	where *A* is the Temkin isotherm energy constant (L/g) and *B* is the Temkin isotherm constant.
Pseudo-first-order model	In(*q*_*e*_ - *q*_*t*_) = In(*q*_*e*_) - *k*_1_*t*	where *q*_*t*_ are the biosorption capacity at time t (mg/g); *k*_*1*_ is the rate constant of first-order biosorption (min^-1^).
Pseudo-second-order model	$\frac{t}{q_{t}}=\frac{1}{k_{2}q_{e}^{2}}+\frac{1}{q_{e}}t$	where *k*_*2*_ is the second-order biosorption rate constant (g/mg min).
Intraparticle diffusion model	*q*_*t*_ = *K*_*id*_*t*^1/2^ + *A*_i_	where *K*_*id*_ is the intraparticle diffusion rate constant (mg/g min^1/2^) and *A*_*i*_ is the intercept.

### Taguchi method

The Taguchi method was used to study the effect of various control factors on the biosorption efficient of MG biosorption from aqueous solution and to determine the significant factors and the optimum levels of experimental factors.

The Experiment design of using the Taguchi method is as follows:

1. The percent removal and biosorption capacity were determined as the quality characteristics to be optimized.

2. Selecting the controllable factors that can be set and maintained and determining their alternative levels. In this paper, the design factors include pH, contact time, biosorbent dose, initial MG concentration and three levels of each factor are represented in Table 2.

3. Determining and designing the orthogonal array. L_9_ (3^4^) orthogonal array experiment was selected and designed as shown in Table 2.

4. A series of experiments were conducted from one to another as the designed orthogonal experiment.

5. Statistical Package for the Social Sciences (SPSS) 18.0 statistical software was used for the range analysis and the analysis of variance (ANOVA) of the data and then to determine the most important controllable factors, which can maximize the percent removal and biosorption capacity, then to select the optimal levels for those factors.

**Table 2:** **The results of Taguchi method experiment (L**_**9**_** (3**^**4**^**) orthogonal array experiment design)**

**Trail NO.**	**Operating factors and their levels**				**Results**	
	**A**	**B**	**C**	**D**	**Percent removal (%)**	**Biosorption capacity (mg/g)**
1	7	5	0.15	80	82.63	22.035
2	7	6	0.2	100	82.34	20.586
3	7	7	0.25	120	82.87	19.889
4	8	5	0.2	120	81.55	24.465
5	8	6	0.25	80	89.58	14.333
6	8	7	0.15	100	81.51	27.171
7	9	5	0.25	100	85.57	17.134
8	9	6	0.15	120	80.82	32.329
9	9	7	0.2	80	85.95	17.191

A: pH; B: contact time (h); C: biosorbent dose (g); D: initial MG concentration (m/l). The results were the mean of three parallel ones.

## Results and discussion

### Discussion

#### Characterization of biosorbent

**Figure 1:**
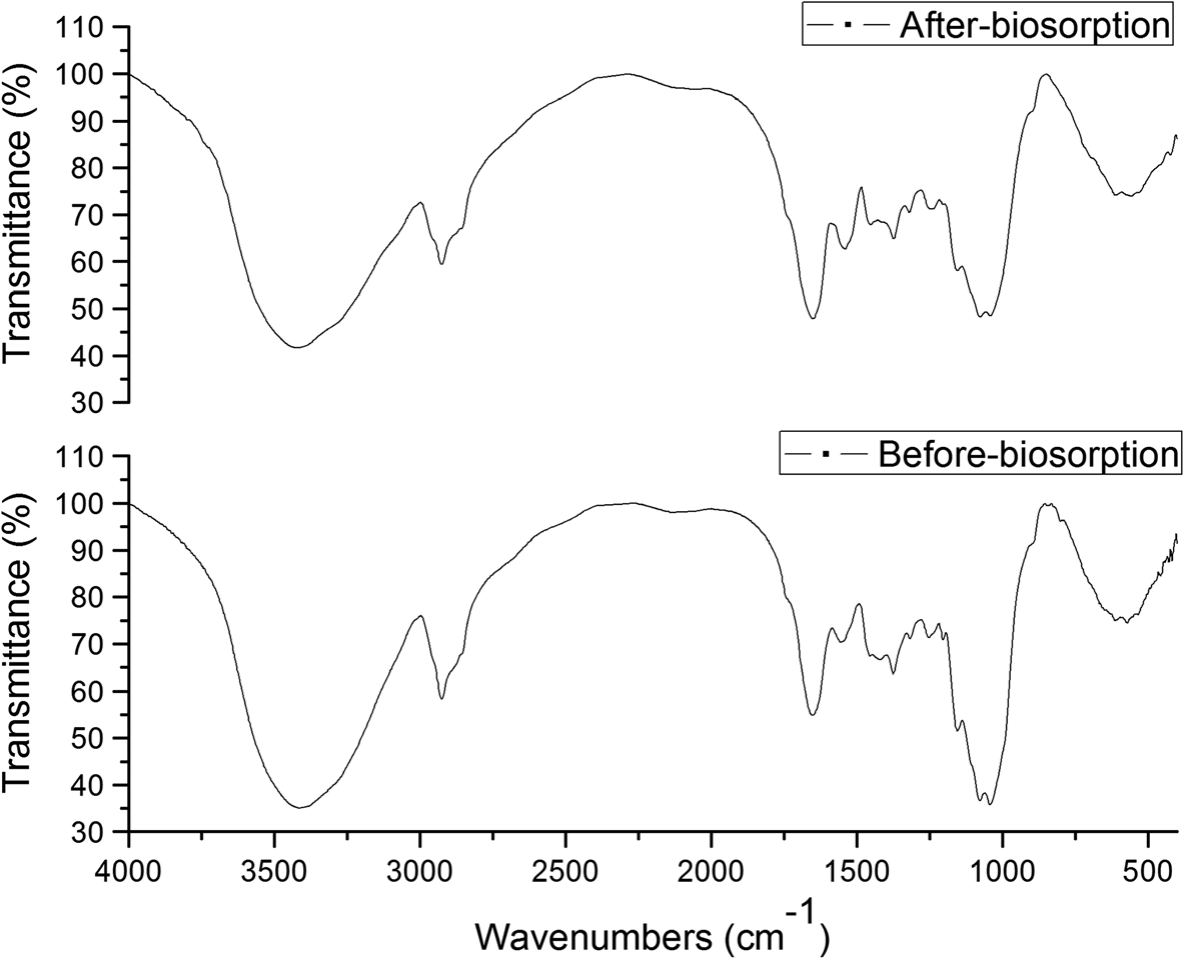
FTIR spectrum of biosorbent (before-biosorption and after-biosorption).

The FTIR spectra of biosorbent that before-biosorption and after-biosorption to confirm the functional groups are responsible for the biosorption process which were shown in Figure 1.

The biosorbent had much more numbers of biosorption peaks before MG biosorption, indicating that a sufficient number of adsorption sites were existed on the surface of biosorbent. For biological biosorbent materials, it always had broad adsorption peak from 3500 to 3200 cm^-1^. The intense peaks at 3500 to 3200 cm^-1^ and 1557 cm^-1^ representing the stretching vibrations of amino groups [30]. The broad peak, which was observed at the level of 3500 to 3200 cm^-1^ for both before-biosorption and after-biosorption, indicated the presence of –OH groups and –N-H groups. The peaks at 2924, 2869 and 2852 cm^-1^ represent the existence of the methyl and methylene groups, and the 2869 cm^-1^ peak disappeared in the FTIR spectrum of after-biosorption biosorbent, indicating that these groups were responsible for the biosorption process. The peaks at 1736 and 1653 cm^-1^ represent the characteristics of carbonyl group stretching from aldehydes and ketones in the FTIR spectrum of biosorbent, and the peak shifted to 1652 cm^-1^ after biosorption [31]. The peak at 1417 cm^-1^ was the characteristics of bending vibration of –OH. The peak at 1375 cm^-1^ was for –CH_3_ stretching vibrations. –C-N stretching vibration of amino acid was observed at the peaks of 1321, 1252 cm^-1^. The peak at around 1153 cm^-1^ was indicative of –P = O stretching vibrations [32]. The peaks at 1083, 1042 cm^-1^ were the characteristics of bending vibration of –C-OH. The peak at 898 cm^-1^ proved that the sulfur in the protein or amino acid also existed. The band between 617 and 536 cm^-1^ for the biosorbent indicated that the existence of –C-N-C scissoring, which can be only found in protein structure. The FTIR spectra of biosorbent indicated that the functional groups such as, carboxyl, hydroxyl, amino and phosphonate groups on the biosorbent surface could be the potential adsorption sites for MG biosorption.

The pHPZC of biosorbent was 6.52, when the pH was below the pHPZC, the biosorbent surface was charged positively to limit the adsorption of MG. Nevertheless, when the pH was above the pHPZC of biosorbent, the surface was charged negative ion to improve the adsorption of MG.

### Effect of biosorbent dose on MG removal

**Figure 2:**
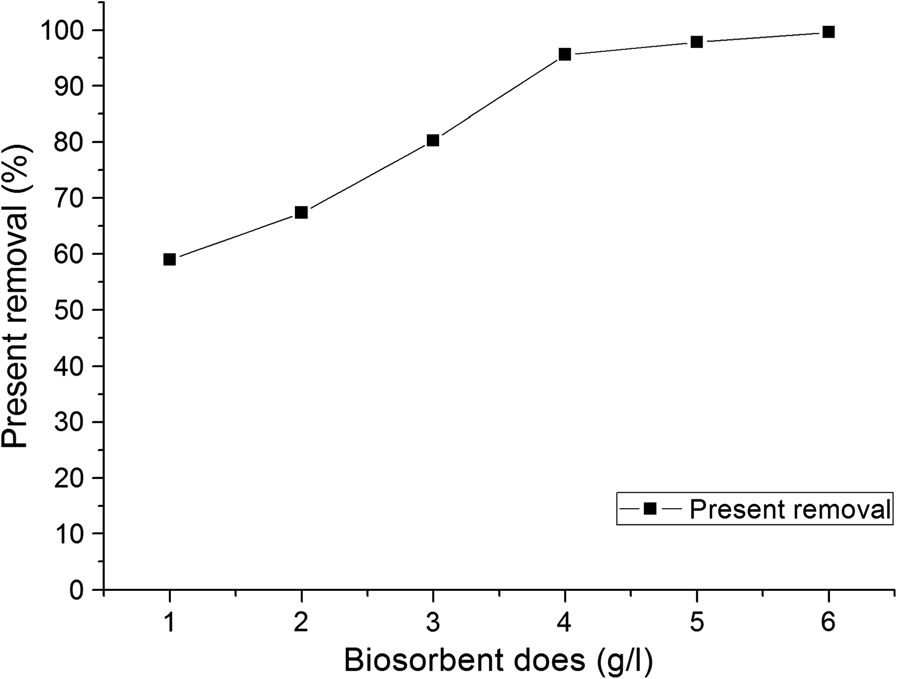
Effect of biosorbent dose on the MG biosorption.

Figure 2 shows that the removal percentage increased with increasing in the biosorbent dose. At equilibrium time of 6 h, the percent removal increased from 58.98% to 99.58% for increasing biosorbent dose from 0.05 to 0.3 g. Above 4 g/L of biosorbent dose, the percent removal of dye MG did not bring about a significant improvement in biosorption at biosorption equilibriums. For economic considerations, optimum biosorbent dose was found to be 4 g/L. The increase of MG removal is owing to the increase of the available biosorption surface areas and availability of more possible biosorption sites with increasing biomass. Similar observations were previously reported for removal of methylene blue from aqueous solution by spent tea leaves [17].

### Effect of pH on MG removal

Dyes in textile industry are complex organic compounds. It is well known that the initial pH of the solution can affect the degree of ionization of the dyes. The surface of the adsorbents is composed of polysaccharides, proteins and lipids with different functional groups (such as amino, carboxyl, thiol and phosphate groups) could bind of the dye molecules. The initial pH affects the structural stability of MG and its color intensity [33]. In the preliminary experiment, it observed by naked eyes that the dye color reduction gradually when adjustment solution pH more than 10 and lower than 2. It may be due to the structural of MG changes being effected at higher pH. MG (pKa = 10.3), the cationic dye, gets protonated in the acidic medium and deprotonated at higher pH. Consequently, the initial pH ranges from 2 to 10 in the experiment.

**Figure 3:**
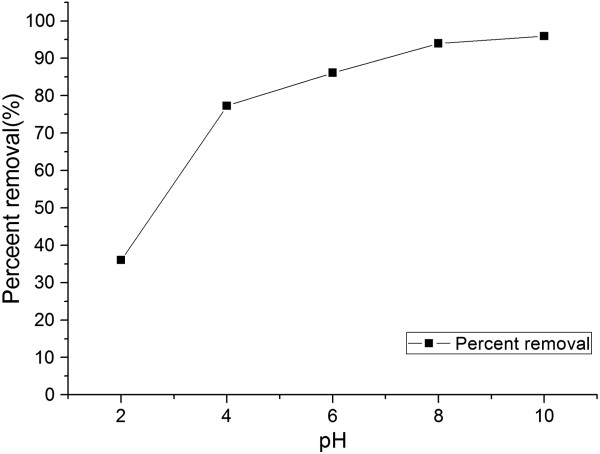
Effect of solution pH on the biosorption of MG.

Figure 3 shows that the percent removal increased gradually from 35.98% to 95.92% with increasing of the pH from 2 to 10, which an increase of nearly 60% of the percent removal. For MG, a cationic dye, existed in aqueous solution in the form of positively charged ion. In addition, the pH_PZC_ of biosorbent was 6.52, when the pH from 2 to 6 which was below the pH_PZC_, the biosorbent surface was charged positively to limit the adsorption of MG. Furthermore, the biosorbent surface was negatively charged when the pH was above the pH_PZC_ of biosorbent [34]. Nevertheless, when the pH value was greater than 6, the removal rate didn’t rise as expected. The pH dependence of dye adsorption was mainly influenced by two factors: firstly, distribution of dye in the solution phase and secondly, overall charge of the adsorbent. A similar result was reported for the biosorption of Neutral Red onto peanut husk [18].

### Effect of ionic strength

There are various impurities, which could be acids, alkalis, salts or metal ions in the wastewaters from textile-manufacturing or dye-producing industries. The presence of cations such as Na^+^, K^+^, Cu^2+^, Ca^2+^ and Cr^3+^, which are the most common metal ions existing in dye wastewater that may affect the performance of the biosorption process.

**Figure 4:**
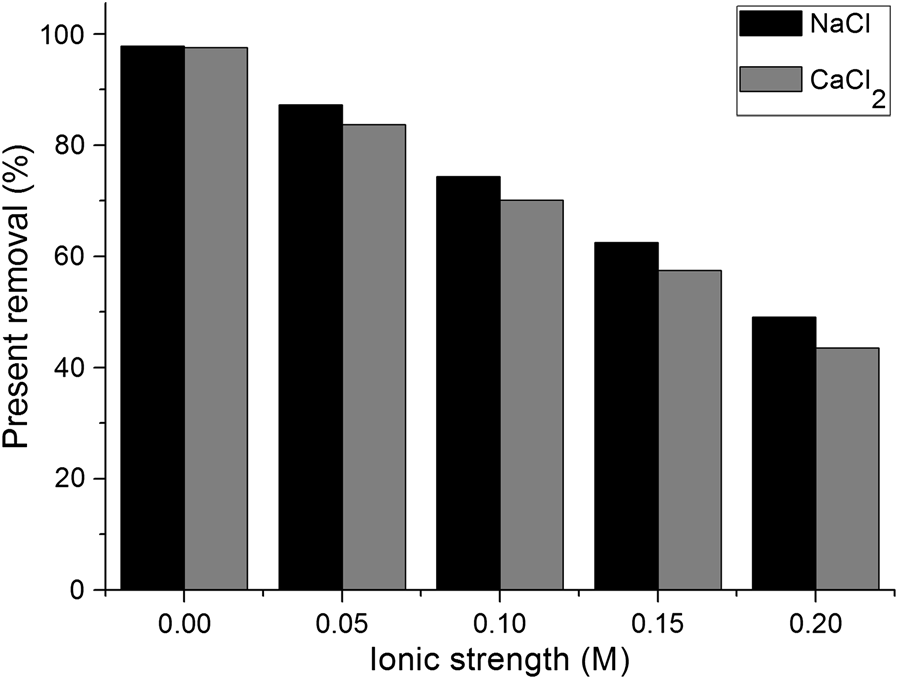
**Effect of Na**^**+**^** and Ca**^**2+**^** on percent removal.**

Figure 4 shows the effect of monovalent Na^+^ and divalent Ca^2+^ on the removal percentage of dye. It was observed that biosorptive capacity for biosorbent decreased with increasing in the concentration of Na^+^ and Ca^2+^. As concentration of Na^+^ changed from 0.05 M to 0.2 M, the percent removal of MG changed from 87.22% to 49.01%, with a decreased of 38.21%. As concentration of Ca^2+^ changed from 0.05 M to 0.2 M, the percent removal of MG changed from 83.73% to 43.57%, with a decreased of 40.16%. From 0.05 to 0.2 M, all the concentration, divalent ions Ca^2+^ had a greater inhibition than monovalent ions Na^+^ on the percent removal, which ranged from 3.49% to 5.44%. With increasing in the concentration, the gap was bigger. It was an adverse effect of ionic strength on MG removal. It may be owed to the possibility of ion exchange mechanisms in the biosorption process. The competition exist between Na^+^, Ca^2+^ and positively charged MG molecules for the same binding sites on the biosorbent surface. Ca^2+^ had greater positive charge than Na^+^ and had a stronger competitive ability. Similar observations were previously reported in aqueous solution for removal of chromium (VI) by Dunaliella species [35] and dye removal by Poly (propylene imine) Dendrimer [36].

### Biosorption isotherms

As seen of Table 3, the regression coefficient *R*^2^ (0.996-0.998) of the linear Freundlich model were higher than the determination coefficient *R*^2^ (0.906-0.972) of Langmuir model and *R*^2^ (0.951-0.956) of Temkin model at all the temperatures, which indicated that Freundlich model was well fitted to data than the Langmuir and Temkin isotherm for this study. Moreover, the maximum MG biosorption capacity (*q*_*m*_) 111.11 mg/g, 125 mg/g, 125 mg/g which got from Langmuir model at 15°C, 25°C, 35°C were found to be much higher compared with the experimental result 24.437 mg/g. These demonstrated that the process of MG biosorption was in terms of Freundlich model, not Langmuir and Temkin model. For Freundlich model, *K*_F_ (l/g) and *n* are Freundlich constants which indicated the ease of biosorption and adsorption intensity, respectively. The value of *n* was bigger than 1, which indicated the biosorption of MG actively and was a heterogeneous nature biosorption and had rapid adsorption process at studied conditions [37]. *K*_F_ and *R*^2^ at 25°C were higher than 15°C, 35°C. The reason could be that with the temperature increased, the molecular of MG movement became faster, more collision frequency between biosorbent and sorbate. It may also be the higher affinity of binding sites on the biosorbent surfaces.

**Table 3:** Isotherm constants for MG biosorption

**Isotherm models**	**Parameters**	**15°C**	**25°C**	**35°C**
*Langmuir*	*q*_*m*_ (mg/g)	111.11	125	125
	*k*_*a*_ (l/mg)	0.00222	0.00204	0.00200
	*R*^2^	0.910	0.972	0.906
*Freundlich*	*K*_F_ (L/g)	0.324	0.336	0.326
	*n*	1.114	1.111	1.107
	*R*^2^	0.996	0.998	0.997
*temkin*	*A*	0.0693	0.0687	0.684
	*B*	10.11	10.63	10.49
	*R*^2^	0.956	0.953	0.951

### Adsorption kinetics

**Figure 5:**
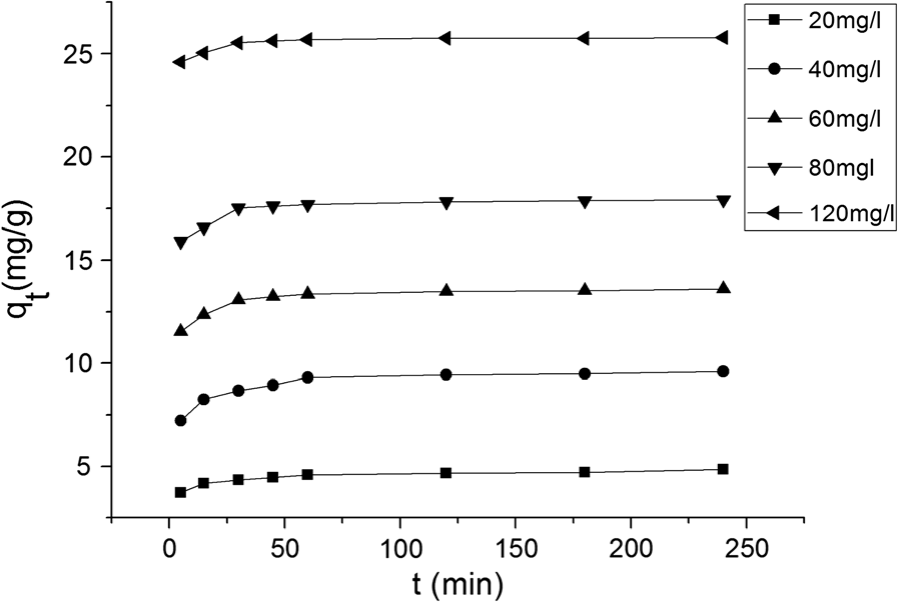
Biosorption at different contact time and different initial MG concentrations.

As seen of Figure 5, the MG biosorption occurred in two stages. In the first stage, it was a rapid uptake within 60 min, then in the second stage, it was slow uptake from 60 to 240 min and reached equilibriumat at 240 min, in this stage, the percent removal of MG did not bring about a significant change.

As seen of Table 4, the *R*^2^ values for pseudo-first-order model was range from 0.912 to 0.989, which was lower than the *R*^2^ values for pseudo-second-order model which ranged from 0.999 to 1.000. Moreover, the theoretical *q*_e,cal_ values for all the studied MG concentration in terms of pseudo-second-order model were more close to the experimental *q*_e,exp_ values compared with the pseudo-first-order model.

**Table 4:** Kinetic parameters for the removal of MG (temperature = 25°C, stirring rate = 180 rpm, pH = 8)

***C***_**0**_**(mg/l)**	***q***_**e,exp**_**(mg/g)**	**Pseudo-first-order model**			**Pseudo-second-order model**		
		***k***_**1**_	***k***_**e1,cal**_**(mg/g)**	***R***^**2**^	***k***_**2**_**(g/mg min)**	***q***_**e2,cal**_**(mg/g)**	***R***^**2**^
20	4.843	0.019	1.369	0.949	0.0412	4.926	0.999
40	9.595	0.021	1.301	0.912	0.0809	9.709	1.000
60	13.604	0.472	2.300	0.989	0.0620	13.699	1.000
80	17.238	0.019	1.141	0.953	0.0570	17.544	1.000
120	24.437	0.017	0.171	0.979	0.4000	25.000	1.000

The initial concentration of MG in the solution remarkably influenced the biosorption. It was noted that initial concentration increased the biosorption of MG (Figure 5). This increase in uptake capacity of the biosorbent with the increase in initial dye concentration is due to higher availability of dye ions for the biosorption [38].

**Figure 6:**
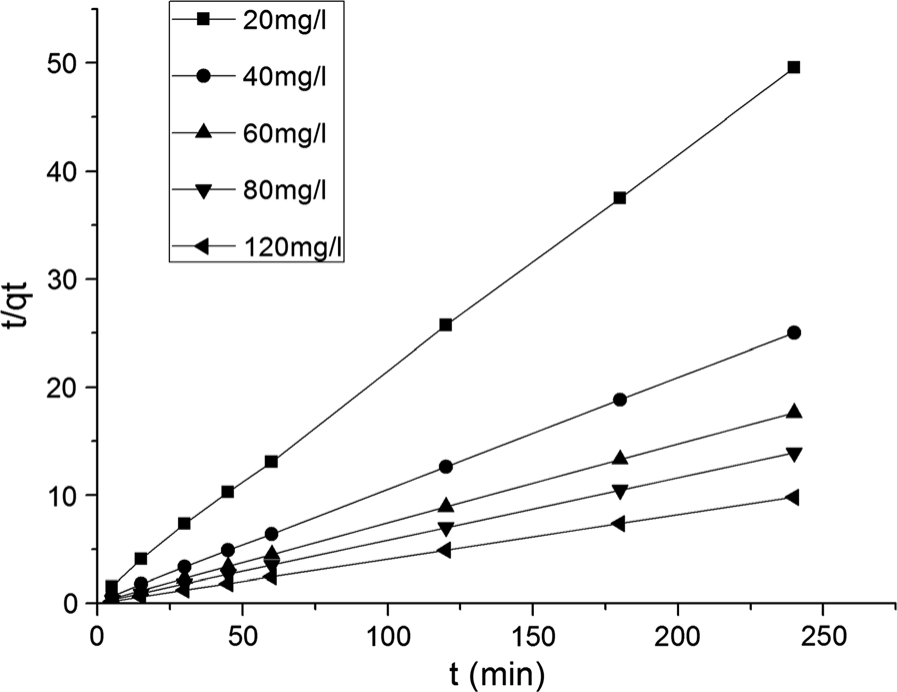
The pseudo-second-order model for MG biosorption at different initial concentrations.

Figure 6 was the linearized plots of the pseudo-second-order kinetic model for MG biosorption. The data of adsorption kinetics were fitted well to the pseudo-second-order linear model for different MG concentration.

**Figure 7:**
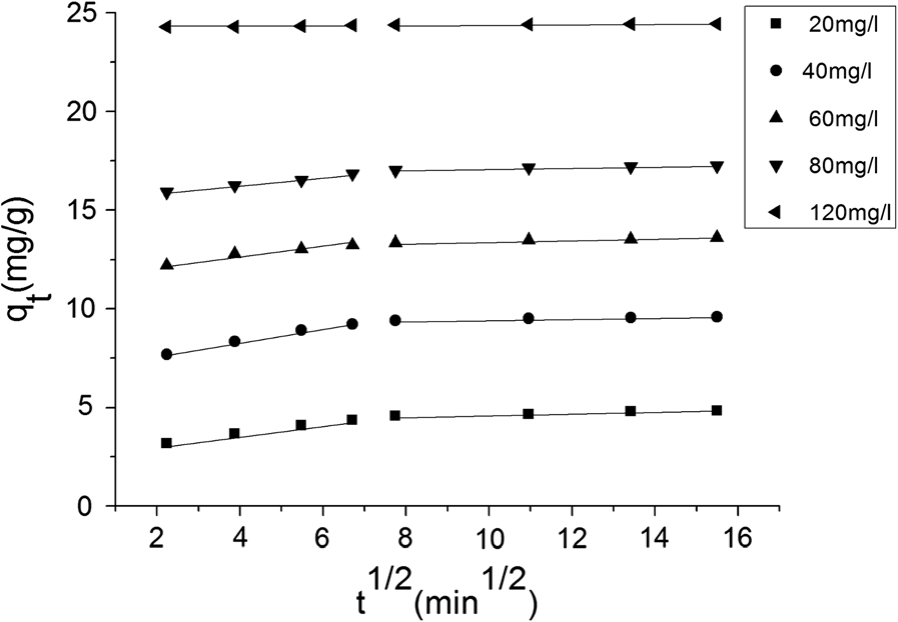
Intraparticle diffusion model for MG biosorption at different initial concentrations.

As showed in Figure 7, the biosorption process was a multi-steps process which included a fast step and a slow step. In the first step, the adsorption active sites on external surface were enough for a fast biosorption. After the first step, the biosorption would get to a steady phase which the intraparticle diffusion was diffusion-controlled.

As seen in Table 5, *K*_id,1_ was larger than *K*_id,2_ which demonstrated the biosorption in the first step got a larger rate of transporting the adsorbed MG from exterior to interior of biosorbent particle. The *A*_i,2_ was bigger than *A*_i,1_ the larger intercept for the greater boundary layer effect, which demonstrated that after a fast biosorption process, most of MG was on the biosorbent, and most of the adsorption active sites on external surface were occupied and made a thick layer. Therefore, the biosorption rate was controlled by the intraparticle diffusion rate. As seen in Figure 7, there were no plots pass through the origin, thus, it demonstrated that intraparticle diffusion was not the only rate control step. Maybe there were other effect such as complexation or ion exchange mechanisms took effect in biosorption process, and the *R*^2^ values for intraparticle diffusion model was ranged from 0.8735 and 0.9957 which were lower than the *R*^2^ values for pseudo-second-order kinetic model.

**Table 5:** Intraparticle diffusion kinetic model (temperature = 25°C, stirring rate = 180 rpm, pH = 8)

***C***_**0**_** (mg/l)**	***K***_**id,1**_** (mg/gmin**^**1/2**^**)**	***A***_**i,1**_	**(*R***_**1**_**)**^**2**^	***K***_**id,2**_** (mg/g min**^**1/2**^**)**	***A***_**i,2**_	**(*R***_**2**_**)**^**2**^
20	0.2671	2.6139	0.9957	0.0350	4.3082	0.9485
40	0.3453	6.9650	0.9878	0.0246	9.2227	0.9467
60	0.2233	11.7936	0.9263	0.0327	13.0999	0.9697
80	0.2041	15.4443	0.9902	0.0282	16.8133	0.9444
120	0.0187	24.2324	0.8735	0.0063	24.3425	0.9753

Many factors influence the adsorption behavior such as structure of dye and adsorbent surface properties. There are many forces which control the adsorption such as van der Waals, hydrogen bonding, steric effect, ion exchange etc. It can be concluded by the results of kinetics that the biosorption process was well in terms of the pseudo-second-order model, which demonstrated that chemisorption was the rate-controlling mechanism through sharing or exchange ions between biosorbent and MG in the biosorption process [39].

### Taguchi method

#### Range analysis (R) of the results of experiment

As seen of Table 2, the trial 5 with the combination of pH 8, contact time 6 h, biosorbent 0.25 g, and initial MG concentration 80 mg/L had the best percent removal and the trial 8 with the pH 9, contact time 6 h, biosorbent 0.15 g, and initial MG concentration 120 mg/L had the best biosorption capacity. Range analysis (R) was used to study the importance of the four factors and get the optimal combination.

The results of R are shown in Table 6. The value of R indicates which factor is more important for the percent removal. K_1_, K_2_, and K_3_ are the average values of the results of the three different levels of each control factor and can determine the optimal level of the specific control factor in Taguchi method experiment. The R was used to estimate the effect of the specific control factor. No matter the value of k_i_ (K_1_, K_2_, and K_3_) or the value of R, all of them comply with the principle of “The larger the better”. As seen, for percent removal, A_K2_,B_K2_,C_K3_,D_K1_ were larger than others results, therefore, the combination of A_2_B_2_C_3_D_1_ which pH 8, contact time 6 h, biosorbent 0.25 g, initial MG concentration 80 mg/L could reach the best percent removal. Through the experimental test, the percent removal reached 89.58%. From Table 6, it can be seen for R_C_>R_D_>R_A_>R_B_, indicating that the biosorbent does was the most important factor on percent removal. For the reason that sufficient biosorbent may afford available sorption surface and availability of more adsorption sites. For biosorption capacity, A_K3_,B_K2_,C_K1_,D_K3_ were larger than others results, therefore, the combination of A_3_B_2_C_1_D_3_ which pH 9, contact time 6 h, biosorbent 0.15 g, initial MG concentration 120 mg/L could have the best biosorption capacity. From Table 6, it can be seen for R_C_>R_D_>R_B_>R_A_, indicating that the biosorbent does was the most important factor on biosorption capacity. For the reason that excessive MG molecule could be fully biosorbed on the surface of the biosorbent. Through the experimental test, the biosorption capacity reached 32.33 mg/g.

**Table 6:** Range analysis of the experiment

	**A**	**B**	**C**	**D**
Percent remove (%)				
K_1_	82.62	83.25	81.65	86.06
K_2_	84.21	84.25	83.18	83.14
K_3_	84.11	83.45	86.01	81.75
R	1.60	1.00	4.35	4.31
Biosorption capacity (mg/g)				
K_1_	20.837	21.204	27.178	17.853
K_2_	21.989	22.416	20.747	21.623
K_3_	22.211	21.417	17.112	25.561
R	1.374	1.212	10.066	7.708

A: pH; B: contact time; C: biosorbent dose; D: initial MG concentration. (The levels were showed in Table 2); K: average value of each level; R: range of K_1_, K_2_ and K_3_.

### Analysis of variance

Analysis of Variance (ANOVA) is a mathematical method which distinguishes the differences between the test results of different experimental conditions and the differences between the test results of causal factors, and the result of ANOVA could obtain which control factor had significantly effect on percent removal and the biosorption capacity [40]. In order to find which factor significantly effect on remove rate and the biosorption capacity, the conditions differences and random differences influenced by any special factor need to be compared. Square sum of deviations were divided by the Corresponding degree of freedom, the reason of the sum of squares were related to the number of observation and the number of groups, therefore, conditions differences and random differences could not compared directly.

In the ANOVA, the Fischer ratio (*F* test) was used to quantify the whole process. The *F* values of each factor were the ratio of the mean of the squared deviations to the mean of the squared error. The critical values for *F* test of confidence level at 0.1 and 0.05 were got in experimental design handbooks. Then the *F* values compared with the critical value at different confidence levels. If the *F* values was larger than *F*_0.05_, indicating that the experiment factor was particularly significant effect on the results and marked with “**”. If *F* value was larger than *F*_0.10_ and smaller than *F*_0.05_, indicating that the experiment factor was significant effect on the results and marked with “*”. At last, if the *F* value was smaller than *F*_0.10_, indicating that the experiment factor had no significant effect on the results.

As seen of Table 7, biosorbent does and initial MG concentration had significant influences on the percent removal. Biosorbent does and initial MG concentration had particularly significant influences on biosorption capacity. The values of *F* were 47.716 and 27.279 for biosorbent does and initial MG concentration, respectively. From the results of ANOVA, it indicated that the biosorption process was not influenced by the time and the environment pH under the condition of this study. In practice, it will show stability in the biosorption process.

**Table 7:** **ANOVA for percent removal and biosorption capacity in the L**_**9**_** (3**^**4**^**) orthogonal array experiment**

**Variance sources**	**SSD**^**a**^	**DOF**^**b**^	**Mean square**	***F*** **ratio**	***F*** **critical value**	**Significance**
Percent removal						
A	4.82 × 10^-4^	2	2.41 × 10^-4^	2.849	F_0.05_(2,2) = 19.0	
B	1.68 × 10^-4^	2	8.40 × 10^-5^	0.996	F_0.10_(2,2) = 9.0	
C	29.00 × 10^-4^	2	14.50 × 10^-4^	17.160		*
D	29.01 × 10^-4^	2	14.51 × 10^-4^	17.163		*
Error	1.69 × 10^-4^	2				
Total	66.20 × 10^-4^	10				
Biosorption capacity						
A	3.267	2	1.634	1	F_0.05_(2,2) = 19.0	
B	2.511	2	1.256	0.769	F_0.10_(2,2) = 9.0	
C	155.903	2	77.951	47.716		**
D	89.127	2	44.564	27.279		**
Error	3.267	2				
Total	254.076	10				

SSD^a^: Sum of Squares of Deviations; DOF^b^: Degree of Freedom.

A: pH; B: contact time; C: biosorbent dose; D: initial MG concentration. The levels were showed in Table 1. Significance levels at 95% and 90% confidence interval, F_0. 05_ (2, 2) = 19, F_0. 10_ (2, 2) = 9.

'*’ indicate the experiment factor was significant effect on the results; '**’ indicate the experiment factor was particularly significant effect on the results.

## Conclusions

This study confirmed that *Pleurotus ostreatus* has the potential to be an economical biosorbent for the removal of MG, a hazardous cationic dye, from wastewater. The biosorption effect in alkaline condition was better than in acidic condition, but pH affected the structural stability of MG and its color intensity when pH was more than 10. MG was almost adsorbed by biosorbent when the initial MG concentration was low in solution. Biosorbent dose, time and pH were important for biosorption in the single factor analysis. Monovalent Na^+^ and divalent Ca^2+^ were found to have an adverse effect on MG removal. Equilibrium time was nearly 240 min. The biosorption equilibrium data were followed the Freundlich isotherm model. The biosorption process was found to follow the pseudo-second-order kinetic model, indicating that chemical biosorption was the rate-controlling mechanism through sharing or exchange ions between biosorbent and MG in the biosorption process.

For the Taguchi method experiment, the optimum condition for percent removal and biosorption capacity were found out. The combination of A_2_B_2_C_3_D_1_ which pH 8, contact time 6 h, biosorbent 0.25 g, initial MG concentration 80 mg/L could reach the highest percent removal 89.58%. And the combination of A_3_B_2_C_1_D_3_ which pH 9, contact time 6 h, biosorbent 0.15 g, initial MG concentration 120 mg/L could reach the largest biosorption capacity 32.33 mg/g. The results of FTIR spectroscopy showed the existence of the functional groups such as, carboxyl, hydroxyl, amino and phosphonate groups on the biosorbent surface.

## Abbreviations

MG: Malachite green; FTIR: Fourier transform infrared; ANOVA: Analysis of variance; SPSS: Statistical Package for the Social Sciences; R: Range analysis; F: Fischer ratio.

## Competing interests

The authors declare that they have no competing interests.

## Authors’ contributions

All authors read and approved the final manuscript.
